# Eosinophilic granulomatosis with polyangiitis resembling Guillain–Barré syndrome under benralizumab treatment

**DOI:** 10.1093/rap/rkae103

**Published:** 2024-08-24

**Authors:** Miyu Wakatsuki, Hiroyuki Yamashita, Ryo Kuwata, Misa Yamaji, Hiroshi Kaneko

**Affiliations:** Division of Rheumatic Diseases, National Center for Global Health and Medicine, Shinjuku, Tokyo, Japan; Division of Rheumatic Diseases, National Center for Global Health and Medicine, Shinjuku, Tokyo, Japan; Division of Rheumatic Diseases, National Center for Global Health and Medicine, Shinjuku, Tokyo, Japan; Division of Rheumatic Diseases, National Center for Global Health and Medicine, Shinjuku, Tokyo, Japan; Division of Rheumatic Diseases, National Center for Global Health and Medicine, Shinjuku, Tokyo, Japan

Key Message

- Eosinophilic granulomatosis with polyangiitis can arise with Guillain–Barré syndrome-like symptoms under benralizumab treatment.

Dear Editor, Benralizumab (anti-IL-5 receptor alpha antibody) is effective against eosinophilic asthma, and it appears efficacious against eosinophilic granulomatosis with polyangiitis (EGPA) [1]. Seven cases of new-onset EGPA during benralizumab treatment have been reported [2]. We describe the first case of EGPA during benralizumab treatment that presented with Guillain–Barré syndrome (GBS)-like symptoms.

An 81-year-old woman with a history of bronchial asthma and eosinophilic pneumonia was hospitalized because of ascending numbness starting from her toes and diarrhoea for a week. Six months prior to admission, her asthma treatment was changed from mepolizumab (100 mg/4 weeks) to benralizumab (30 mg/8 weeks) because of an elevated serum eosinophil count (300/µl) and shortness of breath. Subsequently, the prednisolone (PSL) dose was reduced from 1.5 mg to 0.5 mg/day. On admission to our gastroenterology department 1 month after the last benralizumab dose, blood tests revealed eosinophilia (1251/μl) and elevated CRP levels (3.16 mg/dl). Nerve conduction studies on day 3 of hospitalization revealed multiple motor and sensory mononeuropathies in her legs. Hyponatraemia (117 mEq/l) was also detected. The findings of hypoosmolality (235 mOsm/l), normal plasma vasopressin (1.7 pg/ml), high urine osmolality (347 mOsm/kg), high urine sodium (128 mEq/l) and normal adrenal and kidney function were consistent with syndrome of inappropriate antidiuretic hormone secretion (SIADH). Although cerebrospinal fluid examination revealed no abnormalities, GBS was suspected because of neuropathy, diarrhoea and SIADH [3]. The next day, a blood test revealed a higher eosinophil count (4974/μl), and she was referred to the rheumatology department. At that time, purpura in both lower legs and arthritis of the extremities were noted (Fig. 1A). The other blood test results were as follows: myeloperoxidase-ANCA, <1.0 U/ml; proteinase 3-ANCA, 5.2 IU/ml; CRP, 3.97 mg/dl; T-IgE, 22 615 U/ml; IgG4, 634 mg/dl; IgG, 2341 mg/dl; and negativity for anti-GM-1 IgG and anti-GQ1b IgG. Based on the aforementioned findings, the diagnosis was changed to EGPA (baseline BVAS—version 3 [BVAS] 14). The course of her clinical and laboratory findings is summarized in Fig. 1B. Intravenous immunoglobulin and methylprednisolone pulse therapy, followed by PSL 45 mg, were started on day 4 of hospitalization. The patient’s blood eosinophil count continued to increase after induction therapy but subsequently decreased to 0/μl with treatment, which also led to negativity for CRP and improvement of hyponatraemia. The patient’s lower-limb muscle strength and paraesthesia improved, and she was discharged from the hospital on day 59 with PSL 30 mg. AZA and mepolizumab (300 mg/4 weeks) were introduced to maintain remission. Her disease was inactive (BVAS 3) on PSL 8 mg. On follow-up at 9 months after discharge, her disease was inactive (BVAS 3) on PSL 4 mg.

**Figure 1.**
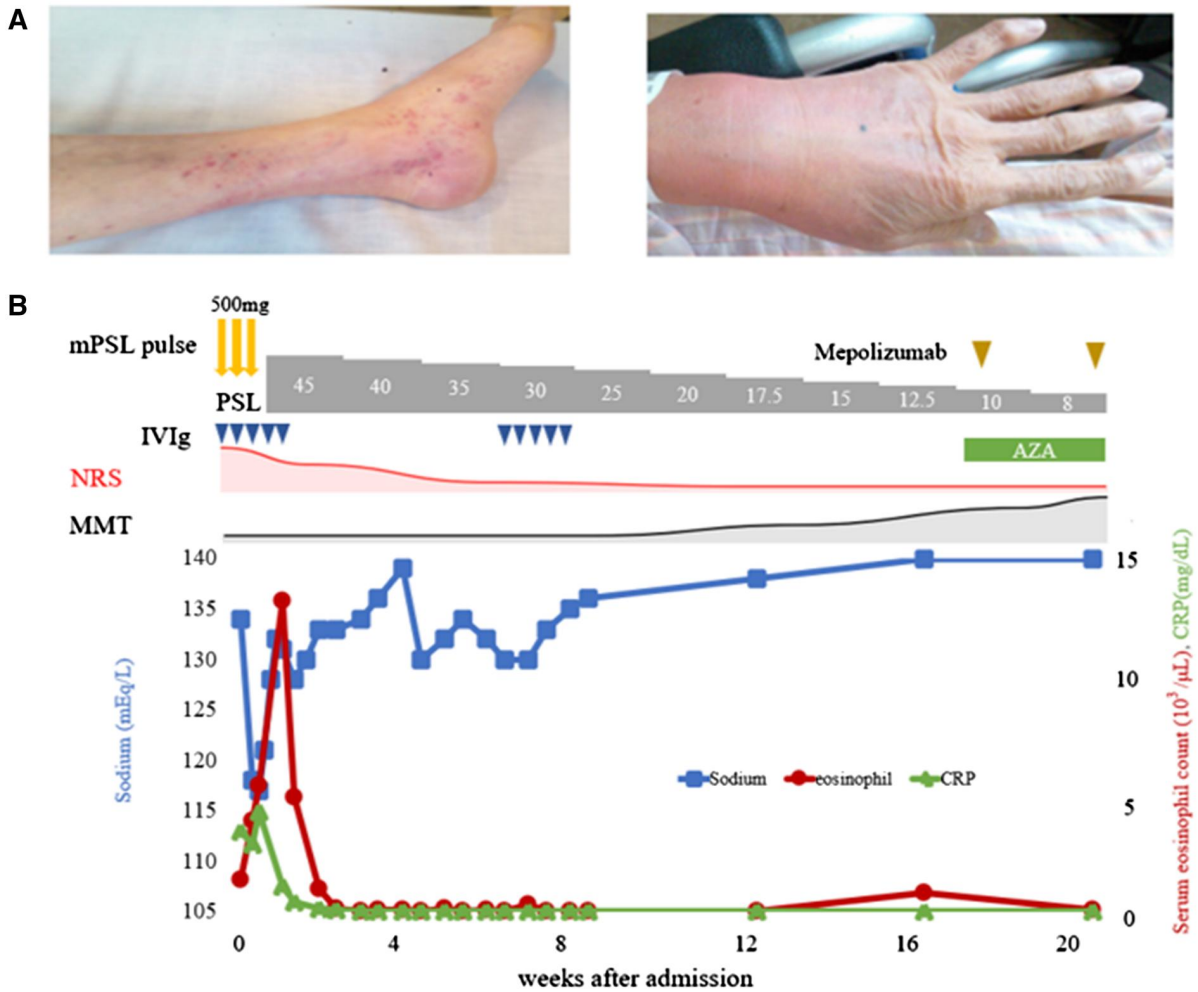
The patient’s presentation at initial examination and clinical course. (A) The patient’s presentation at initial examination (day 4 of hospitalization). Purpura of both lower legs and arthritis of the extremities were noted. (B) The patient’s clinical course. mPSL pulse: methylprednisolone pulse therapy; PSL: prednisolone; NRS: numerical rating scale; MMT: manual muscle testing

To our knowledge, we report the first case of EGPA with GBS-like symptoms under benralizumab treatment. Several reported cases of EGPA were initially diagnosed as GBS because of lower-limb weakness [4], and the time from admission to EGPA diagnosis ranged 3–24 days. Nerve conduction studies revealed sensorimotor axonal polyneuropathy in most patients. In this study, diarrhoea preceded the onset of EGPA, and the patient also had SIADH, resembling GBS [3]. Although our patient had more findings suggestive of GBS than previously described patients, EGPA was diagnosed relatively early (day 4 of hospitalization). Diarrhoea occurs as a gastrointestinal manifestation of EGPA in 23–32% of cases [5]. SIADH is rare in patients with EGPA, and it has been attributed to impaired hypothalamic blood flow secondary to vasculitis or stress-induced antidiuretic hormone hypersecretion [6].

In six of seven previously reported cases [2], the glucocorticoid dose was reduced prior to disease onset because benralizumab was initiated. In the present case, the PSL dose was reduced from 1.5 to 0.5 mg because benralizumab was introduced, suggesting that glucocorticoid dose reduction is associated with the development of EGPA. Another possible mechanism for EGPA development under benralizumab is that the suppression of IL-5 and eosinophils could have masked the onset of EGPA [2]. The clinical course of EGPA is characterized by three phases: prodromal, eosinophilic and vasculitic phases [7]. In this case, the patient previously had eosinophilic pneumonia, and she was considered to be in the eosinophilic phase. Although benralizumab eliminates eosinophils by binding IL-5 receptor alpha, it could not prevent the transition to the vasculitic phase because T1- and T17-related cytokines other than IL-5 are also involved in EGPA development [8]. Additionally, the suppression of eosinophils might have masked signs of EGPA until progression to the vasculitic phase. As evidence, even after induction remission therapy for EGPA in our case, her eosinophil count remained elevated, suggesting the loss of benralizumab efficacy. In patients already in the eosinophilic phase, the use of benralizumab could mask the transition to the vasculitic phase of EGPA by depleting peripheral blood eosinophils.

In conclusion, EGPA can develop with GBS-like symptoms such as neuropathy, diarrhoea and SIADH. Based on reports of EGPA in patients treated with benralizumab, mechanisms other than IL-5 could be involved in its development.

## Data Availability

Data are available upon request.

## References

[1] Wechsler ME , Nair P, Terrier B et al Benralizumab versus mepolizumab for eosinophilic granulomatosis with polyangiitis. N Engl J Med 2024;390:1940. 38810204 10.1056/NEJMc2404353

[2] Yonezawa H , Ohmura SI, Ohkubo Y, Otsuki Y, Miyamoto T. New-onset of eosinophilic granulomatosis with polyangiitis without eosinophilia and eosinophilic infiltration under benralizumab treatment; a case report. Mod Rheumatol Case Rep 2024;8:145–9. 10.1093/mrcr/rxad028 37243733

[3] Anandan C , Khuder SA, Koffman BM. Prevalence of autonomic dysfunction in hospitalized patients with Guillain-Barré syndrome. Muscle Nerve 2017;56:331–3. 28039863 10.1002/mus.25551

[4] Camara-Lemarroy CR , Infante-Valenzuela A, Villareal-Montemayor HJ et al Eosinophilic granulomatosis with polyangiitis presenting as acute polyneuropathy mimicking Guillain-Barre syndrome. Case Rep Neurol Med 2015;2015:981439. 26199772 10.1155/2015/981439 PMC4493297

[5] Gioffredi A , Maritati F, Oliva E, Buzio C. Eosinophilic granulomatosis with polyangiitis: an overview. Front Immunol 2014;5:549. 25404930 10.3389/fimmu.2014.00549 PMC4217511

[6] Dellal FD , Karaahmetli G, Guven SC et al Eosinophilic granulomatosis with polyangiitis as a rare cause of the syndrome of inappropriate antidiuretic hormone secretion. Ir J Med Sci 2023;192:1171–6. 35895178 10.1007/s11845-022-03107-6

[7] Trivioli G , Terrier B, Vaglio A. Eosinophilic granulomatosis with polyangiitis: understanding the disease and its management. Rheumatology (Oxford) 2020;59:iii84–94. 32348510 10.1093/rheumatology/kez570

[8] Hellmich B , Csernok E, Gross WL. Proinflammatory cytokines and autoimmunity in Churg-Strauss syndrome. Ann N Y Acad Sci 2005;1051:121–31. 16126951 10.1196/annals.1361.053

